# Association between Impaired Ketogenesis and Metabolic-Associated Fatty Liver Disease

**DOI:** 10.3390/biom13101506

**Published:** 2023-10-11

**Authors:** Jaehyun Bae, Byung-Wan Lee

**Affiliations:** 1 Division of Endocrinology and Metabolism, Department of Internal Medicine, Catholic Kwandong University College of Medicine, International St. Mary’s Hospital, Incheon 22711, Republic of Korea; 2 Division of Endocrinology and Metabolism, Department of Internal Medicine, Yonsei University College of Medicine, Seoul 03722, Republic of Korea

**Keywords:** ketone, ketogenesis, metabolic-associated fatty liver disease

## Abstract

Metabolic (dysfunction) associated fatty liver disease (MAFLD) is generally developed with excessive accumulation of lipids in the liver. Ketogenesis is an efficient pathway for the disposal of fatty acids in the liver and its metabolic benefits have been reported. In this review, we examined previous studies on the association between ketogenesis and MAFLD and reviewed the candidate mechanisms that can explain this association.

## 1. Introduction

Ketone bodies are synthesized in the liver from free fatty acids (FFAs) and are regarded as thrifty fuels for peripheral tissues [1]. Ketogenesis is considered to be an important part of energy metabolism for survival, especially during long-term fasting, and the ketone bodies present in the body include β-hydroxybutyrate (βHB), acetoacetate, and acetone.

Along with interest in the weight loss effect of ketogenic diets [2], interest in the metabolic significance of ketone bodies or ketogenic pathways has recently increased [2]. While investigating the mechanisms underlying the pleiotropic effects of sodium-glucose cotransporter-2 (SGLT-2) inhibitors, the hypothesis that ketogenesis by SGLT-2 inhibitors is metabolically beneficial has received attention [3,4,5]. Recently, studies on the association between nonpathological spontaneous ketogenesis, which is not caused by certain drugs or diets, and metabolic outcomes, including lower risk of diabetes mellitus and fatty liver disease, have been reported [6,7,8].

Non-alcoholic fatty liver disease (NAFLD) is a liver disease ranging from steatosis to hepatitis, fibrosis, or hepatocellular carcinoma in the absence of excessive alcohol consumption [9]. The definition of “fatty liver” in NAFLD is generally considered to be more than 5% fatty infiltration within hepatocytes, but in most cases, NAFLD is diagnosed clinically through medical history, laboratory measurements, and imaging modalities [10]. NAFLD is currently the most common chronic liver disease [11], with a global prevalence of approximately 30%. Because the development of NAFLD is mostly associated with metabolic dysfunction, the term “metabolic (dysfunction)-associated fatty liver disease” (MAFLD) or metabolic dysfunction-associated steatotic liver disease has been suggested to more accurately reflect its pathogenesis [12,13].

Since hepatic triglyceride (TG) accumulation occurs mainly through the influx of FFAs into the liver or de novo lipogenesis (DNL), and ketogenesis is a type of fatty acid disposal process in the liver, it is expected that ketogenesis and MAFLD are closely related. In addition, given the metabolic benefits of ketogenesis reported in previous studies, the association between ketogenesis and MAFLD is likely to be significant. Studies on the relationship between MAFLD and ketogenesis and its putative mechanisms have been conducted, and although it is still insufficient, the evidence for the relationship is gradually accumulating. In this review, how ketogenesis may affect the development or clinical course of MAFLD is examined, and previous studies on the association between ketogenesis and MAFLD are reviewed.

## 2. Lipid Metabolism in the Pathogenesis of MAFLD and the Ketogenic Pathway

The pathogenesis of MAFLD involves various factors, such as nutritional factors, metabolic dysfunction, genetic susceptibility, and intestinal dysbiosis [14]. These factors have been reported to play respective roles in hepatic steatosis, progression to steatohepatitis, and the development of hepatic fibrosis or cancer. There are several subtypes of MAFLD [15]; however, in general, the main pathological change in the early stage of MAFLD is the accumulation of excess lipids in the liver, regardless of the factors that trigger and exacerbate this process.

### 2.1. Hepatic Lipid Input

There are three main sources of intrahepatic lipid: FFAs from adipose tissue, DNL, and dietary fat [16]. In healthy individuals, FFAs from the adipose tissue account for the highest proportion of lipids supplied to the liver, followed by DNL and dietary fat [17]. This order has been reported to be maintained in patients with MAFLD, but the increase in DNL was most prominent when compared with subjects with low levels of liver fat [17,18].

FFAs from the adipose tissue, the primary source of intrahepatic fat, increase during insulin resistance, which is widely known as a fundamental pathogenic mechanism of MAFLD [19]. Insulin resistance in the adipose tissue induces excess lipolysis, resulting in increased delivery of FFAs to the liver. Along with insulin resistance, expanded or inflamed adipose tissue induced by a Western diet and/or obesity releases large amounts of FFAs into the systemic circulation, which are then delivered to the liver [20,21]. In this way, increased hepatic fatty acids uptake initiates or aggravates MAFLD. Fatty acid transporters such as fatty acid transport proteins and a cluster of differentiation 36 are involved in the hepatic fatty acid uptake [22]. Studies have been conducted on whether the expression of these transport proteins affects the development of MAFLD, but the results are controversial [23,24,25,26].

The second main source of intrahepatic fat, hepatic DNL, is a biochemical process that synthesizes fatty acids from acetyl-CoA subunits derived from various pathways, mainly carbohydrate catabolism [27]. Glucose and fructose are the major substrates of DNL, suggesting that ahigh-carbohydrate diet and hyperglycemia are associated with MAFLD [16,27,28]. The derived acetyl-CoA is converted to malonyl-CoA by acetyl-CoA carboxylase (ACC), and then is converted to palmitate by fatty acid synthase (FAS) [29]. The converted fatty acids enter the process including elongation and esterification, and are synthesized as TGs. In the transcriptional level, DNL process is regulated by sterol regulatory element-binding protein 1c (SREBP1c) and carbohydrate regulatory element-binding protein (ChREBP), activated by insulin, liver X receptor α, and carbohydrate [30,31]. SREBP1c upregulates the expression of ACC and FAS, and was reported to be enhanced in the NAFLD model. A previous study using stable isotopes reported that the contribution of DNL to intrahepatic lipid was significantly higher in subjects with NAFLD than control subjects [17].

Dietary fat is the third most common source of intrahepatic lipid. It can be delivered to the liver via spillover into systemic FFAs or through the uptake of chylomicron remnants [18]. It is estimated that dietary fat accounts for approximately 15% of the intrahepatic lipid.

### 2.2. Hepatic Lipid Output and Ketogenesis

The delivered or synthesized FFAs in the liver undergo a series of steps for TG synthesis [32]. Intrahepatic TGs are then released into the systemic circulation in the form of very low-density lipoprotein (VLDL) particles, which provide fuel for extrahepatic organs. Hepatic steatosis may develop if hepatic TG synthesis exceeds VLDL secretion [33]. In general, the export of TG via VLDL increased with the intrahepatic lipid, but the secretion capacity reaches a plateau when the intrahepatic fat exceeded 10% [34]. After that point, patients with MAFLD cannot secrete additional VLDL, but instead larger, TG-rich VLDL particle. Larger VLDL particles whose diameter exceeds the sinusoidal endothelial pores cannot be secreted and cause fat accumulation in the liver [29,35].

**Figure 1:**
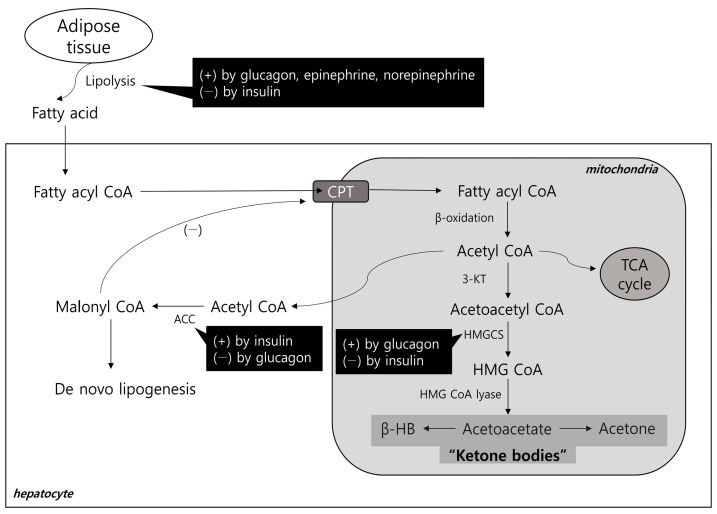
Schematic diagram of the ketogenic pathway and its regulators. (+), activation; (−), inhibition; βHB, β-hydroxybutyrate; 3-KT, 3-ketothiolase; ACC, acetyl-CoA carboxylase; CPT, carnitine palmitoyltransferase (carnitine shuttle); HMGCS, mitochondrial HMG-CoA synthase; TCA, tricarboxylic acid.

Ketogenesis is another process through which FFAs are processed in the liver. This process occurs in the mitochondria of hepatocytes [36] and involves a series of enzymatic reactions (Figure 1). In hepatocytes, FFAs are converted to fatty acyl-CoA, which enters mitochondria via the carnitine shuttle [1,36]. In mitochondria, fatty acyl-CoA undergoes β-oxidation and is metabolized to acetyl-CoA, which is involved in various energy metabolism pathways, such as the tricarboxylic acid (TCA) cycle, lipogenesis, and ketogenesis [36,37].

In the ketogenetic pathway, acetyl-CoA is condensed to acetoacetyl-CoA, which is catalyzed by 3-ketothiolase. Acetoacetyl-CoA is then converted into 3-hydroxy-3-methylglutaryl-CoA (HMG-CoA) by mitochondrial HMG-CoA synthase (HMGCS). This process is the rate-limiting step of ketogenesis. Finally, acetoacetate is formed from HMG-CoA by HMG-CoA lyase. βHB is formed from the reduction of acetoacetate, and acetone is formed by spontaneous decarboxylation of acetoacetate [38]. Acetoacetate and βHB are the prominent forms of ketone bodies, and βHB in particular is the most abundant ketone body in the systemic circulation.

Ketone bodies are efficient fuels that play an important role in survival in glucose-deprived conditions, such as prolonged fasting, and are mainly used by the brain, skeletal muscles, and heart [39,40]. The process of converting ketone bodies into energy is called ketolysis, and this process involves several pathways mediated by enzymes such as succinyl CoA-oxoacid transferase (SCOT) and methylacetoacetyl CoA thiolase [36]. In particular, the reconstitution of acetoacetyl-CoA from acetoacetate by SCOT is the rate-limiting step in ketolysis. Since SCOT activity is very low in the liver, ketolysis occurs mostly in the extrahepatic organs. Ketogenesis is also an effective way to dispose of FFAs when their influx into the liver increases, such as with a high-fat diet and increased lipolysis from adipose tissue [41,42]. Currently, impaired ketogenesis have been reported to be associated with MAFLD, as discussed in Section 3.1 and Section 3.2. In addition, ketone bodies, especially βHB, act as metabolic signaling materials [37], as discussed in Section 3.2.

Nutritional factors and hormones primarily regulate hepatic ketogenesis. As described above, ketogenesis is activated for survival during prolonged fasting but is also induced by a high-fat diet. Similarly, ketogenesis could be induced after exercise, presented as post-exercise ketosis [43,44]. Glucagon, epinephrine, and norepinephrine stimulate lipolysis in adipose tissue [45,46], providing substrates for ketogenesis. In addition, glucagon increases the expression of HMGCS via peroxisome proliferator-activated receptor α (PPARα) [37,46]. These hormones promote ketogenic pathways. In contrast, insulin is a representative hormone that inhibits ketogenesis. It reduces lipolysis in the adipose tissue and suppresses HMGCS expression in the liver [47,48,49]. Insulin also stimulates ACC, which converts acetyl-CoA to malonyl-CoA. Malonyl-CoA inhibits the carnitine shuttle, which inhibits the mitochondrial entry of fatty acids, and thus inhibits ketogenesis. A schematic representation of the ketogenic pathway and its regulators is presented in Figure 1.

## 3. Ketogenesis and MAFLD

### 3.1. Previous Studies on the Association between Ketogenesis and MAFLD

A relatively early study of the association between MAFLD and ketogenesis was published in 1992 by Inokuchi et al. [50]. The study, which began with a question about ketosis resistance in patients with obesity, showed that the plasma total ketone body level, βHB level, and ketone body to FFA ratio were significantly lower in patients with a fatty liver diagnosed by computed tomography than those without a fatty liver.

In 2013, a comparative study reported similar results [51]. Patients who were overweight or obese and had NAFLD showed lower fasting plasma βHB levels than the lean normal population, which was negatively correlated with VLDL cholesterol and plasma TG levels. Another study conducted in 2020 also reported that patients with obesity-related NAFLD had reduced levels of fasting plasma βHB [52].

In 2019, an important study on ketogenesis in patients with NAFLD was published in the United States [53]. The study recruited 40 nondiabetic patients and measured hepatic TG content using proton magnetic resonance spectroscopy (MRS). NAFLD was diagnosed when hepatic TG ≥ 5%. In the study, the fasting plasma ketone levels were lower in patients with NAFLD than in controls. Moreover, when researchers investigated five stable isotope tracers, ketogenesis was progressively impaired as hepatic steatosis worsened and the TCA cycle, an alternative pathway for acetyl-CoA in the mitochondria of hepatocytes, tended to be promoted. This study showed that impaired ketogenesis occurred in NAFLD, and pathways to the TCA cycle were increased, which increased oxidative stress and hepatic glucose production.

Along with growing interest in the ketogenic diet, studies of its therapeutic effects on MAFLD have also been reported [54]. Hepatic steatosis improved even with a short-term (<1 week) ketogenic diet, and a ketogenic diet improved hepatic insulin sensitivity, mitochondrial fluxes, redox state, and hepatic steatosis [55,56].

However, other studies have reported results contrary to those of the studies mentioned above. In 2001, when investigating the pathogenesis of non-alcoholic steatohepatitis (NASH), the fasting plasma βHB levels of each of six patients with NAFLD or NASH diagnosed by liver biopsy were compared with those of a normal control group [57]. Patients with NAFLD or NASH showed higher plasma βHB levels than the normal population. Several years later, studies conducted on nondiabetic subjects also reported higher fasting plasma βHB levels in patients with NAFLD or NASH compared to a normal control group [58,59]. Subsequently, in a slightly larger study recruiting nondiabetic subjects, there was no difference in the plasma βHB levels between patients with NAFLD and normal subjects, which suggested that hepatic lipid oxidation is unchanged in patients with NAFLD, but whole-body lipid oxidation is increased [60].

Recently, several large-scale studies have been published with inconsistent results. In 2021, a large population-based cohort study assessing the association between ketone bodies and NAFLD was published [61]. This study included 6297 participants from a general population-based cohort study. A fatty liver index ≥ 60 was regarded as the cutoff value for NAFLD diagnosis. As a result, fasting plasma ketone body levels, including those of total ketone body, βHB, acetoacetate, and acetone, were higher in patients with NAFLD and were associated with an increased mortality risk. However, studies using spontaneous fasting ketonuria as a marker of ketogenesis reported the opposite trend. Interestingly, these studies were designed to investigate the risk or severity of NAFLD in patients with intact or impaired ketogenesis rather than to examine ketone levels in patients with NAFLD. In 2021, a longitudinal study was published on the risk of incident NAFLD based on the presence of fasting ketonuria in nondiabetic subjects [62]. The study recruited 153,076 nondiabetic patients with no hepatic steatosis and a low probability of fibrosis as assessed by liver ultrasound, NAFLD fibrosis score (NFS), and fibrosis-4 score (FIB-4). During a median follow-up of 4.1 years, subjects with fasting ketonuria showed a lower risk of incident NAFLD, with and without liver fibrosis, and the presence of fasting ketonuria was associated with a lower risk of NAFLD after adjustment for conventional risk factors. Other researchers recruited 6022 nondiabetic patients with NAFLD diagnosed using abdominal ultrasound and compared them with and without fasting ketonuria [63]. In the study, the presence of spontaneous fasting ketonuria in patients was inversely associated with liver fibrosis, as assessed using the NFS and FIB-4, independent of traditional metabolic factors. In 2023, a study on the association between plasma βHB and various noninvasive NAFLD indices in patients with type 2 diabetes was also published [8]. Patients were classified into two groups based on the median fasting plasma βHB level and the intact vs. impaired ketogenesis group. Intact ketogenesis was significantly associated with lower NAFLD indices, especially the NAFLD liver fat score and the Framingham steatosis index.

As reviewed in this section, the results of previous studies on the association between ketogenesis and MAFLD have been inconsistent. The discrepancy between previous studies can be interpreted as reflecting differences in MAFLD status (or stage) and the various metabolic characteristics of the study population. In addition, one hypothesis explaining these inconsistent data is that ketogenesis increases due to increased FFA inflow to the liver in the early stage of MAFLD, thus, from a certain point, the ketogenic pathway is impaired and MAFLD progresses to a more severe status, such as steatohepatitis or fibrosis. A study conducted by Finnish researchers can be cited as a groundwork for this assertion. Data were collected and analyzed for subjects with morbid obesity in normal liver, simple steatosis, and steatohepatitis groups [64]. Interestingly, a significant increase in plasma ketone body levels was observed in the simple steatosis group, whereas ketogenesis decreased in patients with steatohepatitis. Based on the results of recent studies examining the risk and severity of MAFLD according to ketogenic capacity [8,62,63], it can be assumed that ketogenesis is a potential protective mechanism against MAFLD, supporting this hypothesis.

Overall, the results of previous studies are insufficient to conclude that there is an association between ketogenesis and MAFLD, and there have been some limitations. For instance, a considerable number of studies have had limitations in terms of sample size. Additionally, recent large-scale studies have had limitations in terms of MAFLD assessment tools, such as noninvasive indices for MAFLD, which are considered relatively inaccurate compared to MRS or liver biopsy. Table 1 summarizes the studies that have investigated the association between ketogenesis and MAFLD. Large-scale, well-designed studies are required to elucidate the association between ketogenesis and MAFLD.

### 3.2. How Ketogenesis Affects MAFLD

As described above, ketogenesis is a potent and efficient pathway for the disposal of fatty acids in the liver. During the onset and progression of MAFLD, in which lipolysis in adipose tissue, DNL, or dietary fat increases, ketogenesis can be an effective compensatory pathway for the liver to cope with and process heavy fatty acid loads. Because ketogenesis is a nonoxidative pathway and the ketone bodies produced are released into the extrahepatic tissues [65,66], they do not cause additional energy consumption or significant oxidative stress in the liver.

When the ketogenic pathway is impaired, acetyl-CoA, which is produced by β-oxidation, enters the TCA cycle instead, and is oxidized to carbon dioxide. Previous studies using HMGCS knockdown mouse models have reported these changes; in mice lacking HMGCS, hepatic ketogenesis was impaired and TCA cycle flux was increased [67,68]. In addition, hepatic gluconeogenesis and DNL increase because of the intermediates of the TCA cycle. Consistent with these changes, mice lacking HMGCS developed severe hepatic steatosis and hepatitis after a high-fat diet.

In 2019, a study that recruited patients with NAFLD reported results consistent with those of previous animal studies [53]. In the study, ketogenesis was impaired in patients with NAFLD, whereas the alternative TCA cycle was activated, similar to mice lacking HMGCS. In addition, the utilization of acetyl-CoA in the TCA cycle was associated with increased pyruvate carboxylase flux, which represents the anaplerotic pathway of the TCA cycle and is the control point for gluconeogenesis. Therefore, patients with ketogenesis-impaired NAFLD show increased gluconeogenesis and oxidative stress, which may affect glucose homeostasis, provide a substrate for DNL, and cause liver injury, leading to inflammation and fibrotic processes [69].

During the process of ketogenesis, PPARα and its downstream target, fibroblast growth factor 21 (FGF21), are reported to be induced [70,71]. FGF21 is essential for adequate ketogenesis during ketogenic diets [72], and is also known as an important regulator of glucose and lipid metabolism [73,74]. FGF21 has recently been receiving attention as a therapeutic target [75], and has been reported to improve insulin resistance and NAFLD [76,77]. Therefore, induction of FGF21 expression during ketogenesis could be considered to contribute to the protective effects on MAFLD.

In addition to these mechanisms, there is accumulating evidence that ketone bodies can act as signaling substances and have protective effects against MAFLD [78]. βHB, the most prominent form of ketone bodies in systemic circulation, is a ligand for a G-protein-coupled receptor named hydroxycarboxylic acid receptor 2 (HCAR2) [79]. When βHB binds to adipocyte-expressing HCAR2, adipocyte lipolysis is suppressed. Through HCAR2 activation, βHB also improves insulin resistance by regulating peroxisome proliferator-activated receptor γ-related genes [80]. This is a mechanism in line with the results of previous studies in which the concentration of FFA and glucose in the bloodstream decreased when βHB was administered to animals or humans [81,82,83,84]. Through the HCAR2-mediated pathway, βHB downregulates lipogenesis-related genes such as ACC and FAS and upregulates AMP-activated protein kinase, which inhibits hepatic endoplasmic reticulum (ER) stress and lipid accumulation [85]. In addition, βHB binds to FFA receptor 3 (FFAR3), responsible for the sympathetic tone [86]. As an antagonist, it suppresses sympathetic activity via FFAR3. βHB is also involved in inflammatory pathways. It inhibits reactive oxygen species-induced inflammation, ER stress and NOD-, LRR-, and pyrin domain-containing protein 3 inflammasome-mediated inflammatory responses [87,88,89]. Increased sympathetic tone and inflammation are important factors in the pathogenesis of MAFLD [90,91], and these actions of βHB are believed to protect against the progression of MAFLD. Recently, based on the various action mechanisms and effects of βHB, βHB has received attention as a therapeutic target for various liver diseases and metabolic diseases, including MAFLD [84,92].

However, most studies that have suggested the role of βHB as a signaling or regulating material have been in vitro or animal studies. Therefore, clinical evidence is still lacking and a large number of related clinical studies are needed.

## 4. Conclusions

Ketogenesis is an effective, potent, and safe method for the disposal of fatty acids in the liver. When the ketogenic pathway is impaired, the TCA cycle is activated as an alternative pathway, and gluconeogenesis, DNL, and oxidative stress increase, which are thought to induce the development and/or aggravation of MAFLD. In addition, βHB, one of the ketone bodies, has been reported to have inhibitory effects on adipocyte lipolysis, intrahepatic fat accumulation, and inflammatory responses, showing the possibility of having a protective effect on MAFLD. Although the results of previous studies on whether ketogenesis decreases (or increases) in patients with MAFLD are inconsistent, considering the mechanistic aspects, ketogenesis is considered to have a protective effect against MAFLD.

The discrepancy between previous studies may reflect the complexity of metabolic pathways or disorders including ketogenesis and MAFLD, but may also be due to methodologic issues such as an insufficient sample size and inaccurate assessment of MAFLD status. Large-scale, well-designed, or longitudinally tracked studies that accurately reflect the severity of MAFLD are required to elucidate the association between ketogenesis and MAFLD.

## Figures and Tables

**Table 1:** Studies reporting the association between ketogenesis and MAFLD.

Reference	Subjects	Independent Variable	Dependent Variable	Main Finding
Inokuchi et al. [50]	20 NGT patients with obesity	NAFLD by computed tomography	Fasting plasma total ketone, βHB	Low fasting total ketone, βHB in NAFLD
Croci et al. [51]	15 lean healthy, 20 NAFLDsubjects with overweight or obesity	NAFLD by liver biopsy	Fasting plasma βHB	Low fasting plasma βHB in NAFLD
Männistö et al. [64]	76 patients with obesity	Steatosis by ^1^H-MRS, NASH by liver biopsy	Fasting plasma βHB, acetoacetate	Increased ketone bodies in simple steatosis but decreased in NASH
Fletcher et al. [53]	40 non-diabetic patients	NAFLD by ^1^H-MRS	Fasting plasma ketone, ketogenic pathway measured by 5 stable isotope tracers	Low fasting ketone and ketogenesis in NAFLD
Mey et al. [52]	22 patients with obesity	NAFLD by ^1^H-MRS	Fasting plasma βHB	Low fasting plasma βHB in NAFLD
Kim et al. [62]	153,076 nondiabetic subjects	Fasting ketonuria	Steatosis by ultrasound Fibrosis by NFS and FIB-4	Low risk of NAFLD in subjects with ketonuria
Lim et al. [63]	6022 nondiabetic NAFLD patients	Fasting ketonuria	Fibrosis by NFS and FIB-4	Low risk of advanced fibrosis in subjects with ketonuria
Lee et al. [8]	435 type 2 diabetic patients	Fasting plasma βHB	Non-invasive NAFLD indices	Low risk of NAFLD indices in subjects with intact ketogenesis
Kotronen et al. [60]	58 nondiabetic patients	NAFLD by ^1^H-MRS	Fasting plasma βHB	Comparable between NAFLD and control
Sanyal et al. [57]	6 NAFLD, 6 NASH, 6 lean healthy subjects	NAFLD by liver biopsy	Fasting plasma βHB	High fasting βHB in NAFLD and NASH
Chalasani et al. [59]	37 nondiabetic patients	NASH by liver biopsy	Fasting plasma βHB	High fasting βHB in NASH
Bugianesi et al. [58]	18 non-obese, non-diabetic patients	NAFLD by liver biopsy	Fasting plasma βHB	High fasting βHB in NAFLD
Post et al. [61]	6297 general population	NAFLD by FLI score	Fasting ketone bodies, including total, βHB, acetoacetate, acetone	High fasting ketone bodies in NAFLD

βHB, β-hydroxybutyrate; FIB-4, fibrosis-4 score; FLI, fatty liver index; ^1^H-MRS, proton magnetic resonance spectroscopy; NAFLD, non-alcoholic fatty liver disease; NASH, non-alcoholic steatohepatitis; NFS, NAFLD fibrosis score; NGT, normal glucose tolerance.

## Data Availability

No new data were created or analyzed in this study. Data sharing is not applicable to this study.
